# Monitoring and treatment patterns of von Hippel-Lindau disease-associated central nervous system hemangioblastomas

**DOI:** 10.1186/s13053-026-00337-y

**Published:** 2026-04-20

**Authors:** Eric Jonasch, Yan Song, Jonathan Freimark, Manasi Mohan, James Signorovitch, Murali Sundaram

**Affiliations:** 1 The University of Texas MD Anderson Cancer Center, 1515 Holcombe Blvd, Houston, TX 77030 USA; 2 Analysis Group, Inc., 111 Huntington Ave, Boston, MA 02199 USA; 3 Merck & Co., Inc., Rahway, NJ USA

**Keywords:** Pain management drug use, Central nervous system hemangioblastoma, Disease monitoring, Specialist visits, Treatment patterns, von Hippel-Lindau disease

## Abstract

**Background:**

Patients with von Hippel-Lindau-associated central nervous system hemangioblastoma (VHL-CNS-Hb) have a large economic burden, but there is limited real-world evidence describing their clinical burden in the United States (US). This study compared treatment patterns and monitoring procedures between patients with VHL-CNS-Hb and matched controls in the US.

**Methods:**

Patients with VHL-CNS-Hb and matched controls were identified from Optum’s de-identified Clinformatics^®^ Data Mart Database. The index date was the date of first CNS-Hb diagnosis (VHL-CNS-Hb cohort) or the date of a random medical claim within the patient’s claims (control cohort). Treatment patterns were assessed in the VHL-CNS-Hb cohort using unadjusted incidence rates. Pain management drug use, disease monitoring procedures, and visits to medical specialists were compared between cohorts using unadjusted and adjusted generalized linear models. Clinical trial number: not applicable.

**Results:**

The most common treatment observed in the VHL-CNS-Hb cohort (*N* = 220) was targeted therapy for renal cell carcinoma (2.34 treatment events per 10 person-years). Pain management drug use, monitoring, and specialist visits peaked during the first/second year post-index. Compared to controls (*N* = 1100), VHL-CNS-Hb patients had higher rates of pain management drug use (adjusted incidence rate ratio [IRR]: 1.96), monitoring procedures (adjusted IRR: 3.93), and specialist visits (adjusted IRRs: 2.49–25.44; all *P* ≤ 0.001).

**Conclusions:**

Patients with VHL-CNS-Hb had a substantial, long-term clinical burden that included treatment for multiple VHL manifestations—reflecting the multi-organ nature of the disease—frequent pain management drug use, and high levels of healthcare use for disease monitoring and specialist visits.

## Background

As an inherited tumor syndrome, von Hippel-Lindau (VHL) disease is characterized by the development of benign and malignant tumors in multiple organ systems [1]. Approximately 7,001 individuals were affected by VHL disease in the United States (US) in 2019, with an estimated prevalence rate of 2.13 per 100,000 [2]. 

The first manifestation of VHL disease is often central nervous system hemangioblastoma (CNS-Hb), but other lesions may include renal cell carcinomas (RCCs), pheochromocytomas, pancreatic neuroendocrine tumors, and retinal Hbs [1]. CNS-Hb develops in approximately 60% to 80% of patients with VHL disease and is commonly multifocal, with the cerebellum being the most frequently affected site [1, 3]. While CNS-Hbs are typically benign, patients may experience symptoms caused by the tumor’s mass effect on adjacent regions, such as headaches, nausea, and vomiting [3]. Notably, CNS-Hb is the primary cause of mortality among patients with VHL disease, representing half of all deaths [3, 4]. 

Management of VHL-associated CNS-Hb (VHL-CNS-Hb) is mainly centered on surgery for symptomatic tumors [5]. While regular screening and surveillance is recommended for all patients with VHL disease, post-operative surveillance is particularly important and may be more frequent given the risk of complications like neurological deficits [1, 4, 6]. The widespread nature of VHL manifestations means that patients often have multiple operations over their lifetime, thus requiring long-term care from a multidisciplinary team of specialists, including neurosurgeons, urologic oncologists, and ophthalmologists [7, 8]. As such, patients with VHL-CNS-Hb incur high rates of healthcare resource utilization and costs, particularly for surgical removal of CNS-Hb [9]. Indeed, in a recent claims-based study conducted in the US, patients with VHL-CNS-Hb were observed to have significantly more inpatient, outpatient, and emergency department visits than control patients without VHL disease or CNS-Hb, which translated to $49,645 higher annual healthcare costs [9]. 

Given the large economic burden of VHL-CNS-Hb, there is a need to gain a better understanding of the associated clinical burden, including the treatment patterns of patients with VHL-CNS-Hb and the healthcare resources used for VHL disease monitoring. However, there is currently limited real-world evidence of patients with VHL-CNS-Hb in the US [9, 10]. Therefore, the current study was conducted to expand upon the prior economic burden analysis by describing and comparing the use of treatments and pain management drugs, as well as disease monitoring procedures and specialist visits between patients with VHL-CNS-Hb and matched control patients in the US.

## Methods

### Data source

Administrative claims data from 2007 to 2020 from Optum’s de-identified Clinformatics^®^ Data Mart Database (CDM) were used. The CDM is a single-payer source of US claims data for more than 60 million lives from all 50 states, predominantly composed of patients from the South and North Central (Midwest) census regions. All patients aged ≥ 65 years are insured by commercial or Medicare Advantage plans.

Since data were de-identified and comply with the patient requirements of the Health Insurance Portability and Accountability Act of 1996, no reviews by an institutional review board were required.

### Study design

A retrospective cohort design was used to describe and compare treatment patterns and disease monitoring between patients with VHL-CNS-Hb and their matched controls. The index date was defined as the date of the first observed CNS-Hb diagnosis for the VHL-CNS-Hb cohort or the date of a random medical claim within the patient’s claims history for the control cohort. The baseline period was defined as the 12-month period prior to the index date. The study period comprised the time between the index date and the earliest of the end of continuous healthcare coverage, end of data availability, or up to 5 years.

### Sample selection

**Fig. 1:**
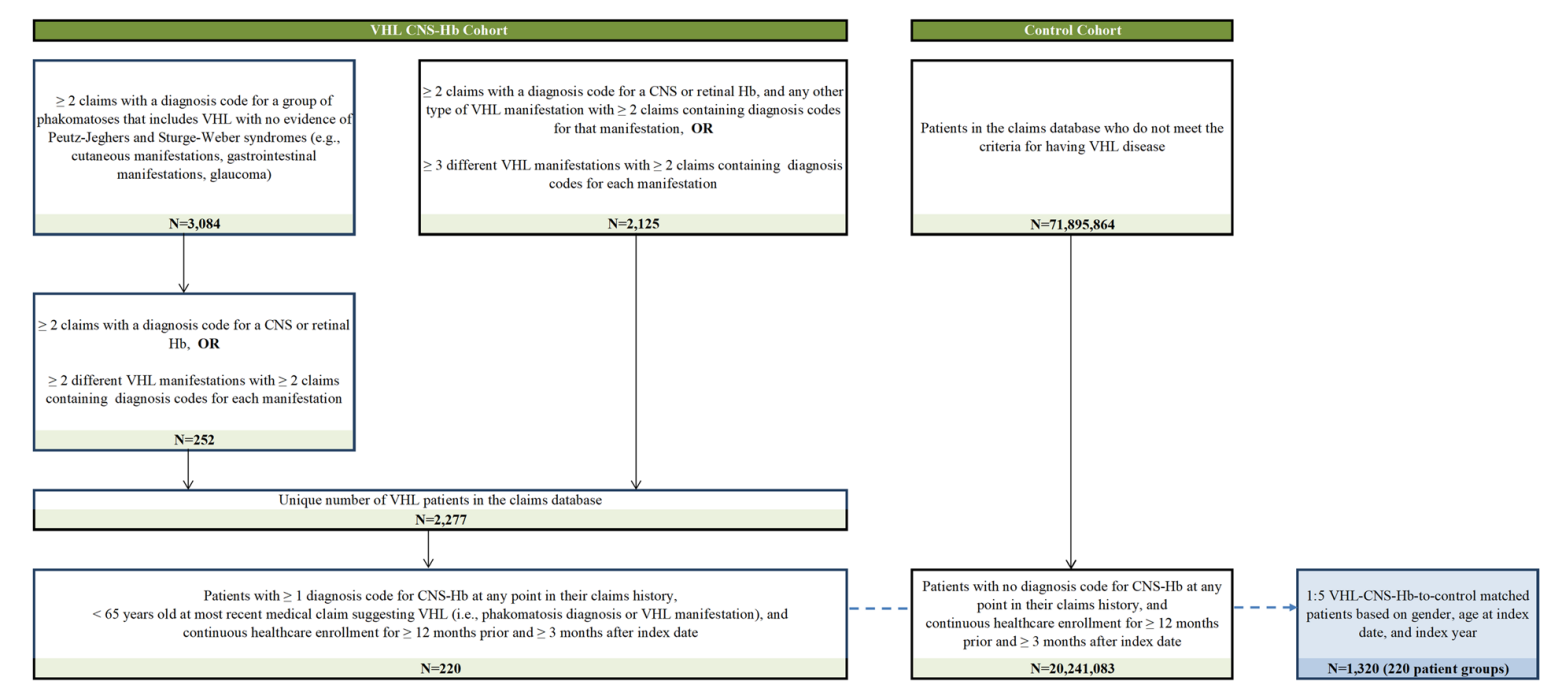
Sample selection of patient cohorts. **Abbreviations**: CNS: central nervous system; Hb: hemangioblastoma; VHL: von Hippel-Lindau

There was no International Classification of Diseases (ICD) diagnosis code for VHL disease until recently; therefore, a previously developed and validated claims-based algorithm was used to identify patients with VHL disease in this study (Fig. 1) [2, 9]. Briefly, patients were identified as having VHL disease based on a diagnosis of phakomatosis or diagnoses of VHL disease manifestations; additional details are available in the prior studies [2, 9].

Patients with VHL disease were further identified as having VHL-CNS-Hb if they had ≥ 1 diagnosis code for CNS-Hb at any point in their claims history. These patients were required to be aged < 65 years at the most recent medical claim suggesting VHL disease (i.e., phakomatosis diagnosis or VHL disease manifestation) to reduce the chance of capturing patients with sporadic tumors that were not associated with VHL. Patients in the control cohort did not meet the aforementioned criteria for having VHL disease and had no diagnosis code for CNS-Hb at any point in their claims history. Additionally, patients in both cohorts were required to have continuous healthcare enrollment for ≥ 12 months prior to and ≥ 3 months following the index date. Patients in the VHL-CNS-Hb cohort were matched 1:5 to control patients using propensity score based on age at index date, sex, and index year.

### Study outcomes

Study outcomes included treatment patterns in the VHL-CNS-Hb cohort and pain management drug use, disease monitoring procedures, and visits to medical specialists in both cohorts.

Treatment patterns for patients in the VHL-CNS-Hb cohort were assessed during the study period and included unadjusted incidence rates (as events per 10 person-years) of nephrectomy, renal ablation, radiotherapy, targeted therapy for RCC (i.e., axitinib, belzutifan, bevacizumab, cabozantinib, everolimus, lenvatinib, nivolumab, pazopanib, pembrolizumab, sorafenib, sunitinib), retinal laser therapy, and surgical removal of cerebellar and spinal Hbs, adrenal pheochromocytomas, or pancreatic neuroendocrine tumors. The list of treatments included therapies and procedures used to treat additional manifestations of VHL beyond CNS-Hb, as patients with VHL commonly develop tumors across organ systems.

Pain management drug use included opioids, non-steroidal anti-inflammatory drugs (NSAIDs), corticosteroids, cannabinoids, anti-epileptics, anti-depressants, alpha-2 adrenergic agonists, and botulinum toxin. These drug classes, while not all strictly analgesics, were included as they are commonly used for pain management, including for neuropathic pain management [11]. Disease monitoring procedures included ultrasounds, magnetic resonance imaging (MRI), computerized tomography (CT) scans, chest x-rays, retinal exams, metanephrines tests, and renal function tests. Visits to medical specialists included oncology, urology, nephrology, genetics, neurosurgery, ophthalmology, and endocrinology visits. All events were measured in both cohorts as unadjusted and adjusted incidence rates. All incidence rates were reported as events per person-year and were assessed over the entire study period. Separately, to observe trends over time, unadjusted incidence rates of pain management drug use, disease monitoring procedures, and visits to medical specialists were measured yearly from 2 years pre-index to 5 years post-index among patients in the VHL-CNS-Hb cohort who had ≥ 6 months of continuous eligibility in each given year.

### Statistical analysis

Baseline variables were compared between the VHL-CNS-Hb and control cohorts using univariable generalized estimating equation (GEE) models. For the comparison of incidence rates of pain management drug use, disease monitoring procedures, and visits to medical specialists between the cohorts, unadjusted and adjusted generalized linear models were fit with a negative binomial distribution and log-link function. A GEE model was used to account for correlation within matched pairs. The regression models adjusted for age at index, sex, and the number of outpatient visits at baseline. Unadjusted and adjusted incidence rate ratios (IRRs) were reported, along with the corresponding 95% confidence intervals (CIs) and P-values.

## Results

### Baseline characteristics

The study included 220 patients in the VHL-CNS-Hb cohort and 1,100 patients in the control cohort. Baseline characteristics were previously described by Jonasch et al. [9]. Briefly, mean age was 49.0 years in both cohorts and 38.2% and 38.4% were male in the VHL-CNS-Hb and control cohorts, respectively (Table 1). Patients in the VHL-CNS-Hb cohort had significantly higher rates of comorbidities, healthcare resource utilization, and healthcare costs than matched patients in the control cohort (all *P* < 0.05).

**Table 1:** Baseline characteristics of patients with VHL-CNS-Hb and control patients

	VHL-CNS-Hb Patients	Control Patients	*P*-value	
	(*N* = 220)	(*N* = 1,100)		
**Demographic characteristics**				
Male, N (%)	84 (38.2%)	422 (38.4%)		
Age at index date (years), mean ± SD	49.0 ± 11.3	49.0 ± 11.2	-	
Geographic region, N (%)			0.958	
Northeast	22 (10.0%)	107 (9.7%)		
Midwest	51 (23.2%)	284 (25.8%)		
South	108 (49.1%)	479 (43.5%)		
West	38 (17.3%)	220 (20.0%)		
Unknown	1 (0.5%)	10 (0.9%)		
Health insurance type, N (%)			0.633	
EPO	18 (8.2%)	108 (9.8%)		
PPO	17 (7.7%)	42 (3.8%)		
POS	121 (55.0%)	723 (65.7%)		
Indemnity	0 (0.0%)	1 (0.1%)		
HMO	43 (19.5%)	184 (16.7%)		
Other^1^	21 (9.5%)	42 (3.8%)		
Year of index date, N (%)				
2008–2011	67 (30.5%)	335 (30.5%)	-	
2012–2015	71 (32.3%)	355 (32.3%)	-	
2016–2020	82 (37.2%)	410 (37.2%)	-	
**Comorbidities**				
Charlson Comorbidity Index, mean ± SD	2.0 ± 2.6	0.4 ± 1.0	< 0.001	*
Comorbidities, N (%)				
Hypertension	100 (45.5%)	327 (29.7%)	< 0.001	*
Depression/anxiety	73 (33.2%)	181 (16.5%)	< 0.001	*
Chronic pulmonary disease	54 (24.5%)	103 (9.4%)	< 0.001	*
Cerebrovascular disease	51 (23.2%)	27 (2.5%)	< 0.001	*
Cancer	50 (22.7%)	43 (3.9%)	< 0.001	*
Liver disease	37 (16.8%)	33 (3.0%)	< 0.001	*
Diabetes	34 (15.5%)	108 (9.8%)	0.032	*
Osteoporosis/fractures	30 (13.6%)	59 (5.4%)	< 0.001	*
Renal disease	24 (10.9%)	20 (1.8%)	< 0.001	*
Peripheral vascular disease	19 (8.6%)	22 (2.0%)	< 0.001	*
Myocardial infarction	9 (4.1%)	8 (0.7%)	0.013	*
**Annual healthcare resource utilization during baseline**,** mean ± SD**				
Inpatient visits	0.73 ± 1.67	0.14 ± 0.60	< 0.001	*
Length of inpatient visits	2.88 ± 7.44	0.78 ± 7.46	< 0.001	*
Emergency department visits	1.84 ± 4.68	0.55 ± 1.76	< 0.001	*
Outpatient visits	15.15 ± 10.62	7.67 ± 8.77	< 0.001	*
Other visits^2^	1.75 ± 2.95	0.83 ± 2.17	< 0.001	*
**Annual healthcare costs during baseline**^**3**^, **mean ± SD**				
Total costs	$44,688 ± $92,113	$9,102 ± $40,331	< 0.001	*
Medical costs	$38,869 ± $87,536	$7,371 ± $39,206	< 0.001	*
Inpatient costs	$21,270 ± $75,640	$3,435 ± $34,478	< 0.001	*
Emergency department costs	$3,600 ± $12,781	$635 ± $2,579	< 0.001	*
Outpatient costs	$13,519 ± $36,804	$3,122 ± $11,500	< 0.001	*
Other costs^2^	$480 ± $2,513	$178 ± $1,685	0.087	
Pharmacy costs	$5,819 ± $22,709	$1,731 ± $7,090	0.008	*

**Abbreviations**: CCI: Charlson Comorbidity Index; CNS: central nervous system; EPO: exclusive provider organization; GEE: generalized estimating equation; Hb: hemangioblastoma; HMO: health maintenance organization; ICD-9/10-CM: International Classification of Diseases, Ninth/Tenth Revision, Clinical Modification; POS: point of service; PPO: preferred provider organization; SD: standard deviation; USD: United States dollars; VHL: von Hippel-Lindau

**Notes**:

* denotes P-value < 0.05

[1] The “other” health insurance category includes the following types of plans: independent practice organizations, lock-ins, pharmacy network plans, unknown, and no coverage

[2] “Other” medical visits include all those not captured by inpatient, emergency department, or outpatient costs. These claims may reflect home health aids or physician visits among other types of ancillary medical claims

[3] Cost values are inflated to 2020 USD using Consumer Price Index data from the US Bureau of Labor Statistics

### Treatment patterns

Among patients in the VHL-CNS-Hb cohort, the most common treatment observed during the study period was targeted therapy for RCC, with an incidence rate of 2.34 treatment events (i.e., prescriptions or refills for oral medications and administrations of intravenous medications) per 10 person-years (Table 2). Common targeted therapies for RCC included bevacizumab (0.73 per 10 person-years), pazopanib (0.62 per 10 person-years), and cabozantinib (0.33 per 10 person-years). The incidence rate of surgical removal of cerebellar and spinal Hbs was 0.52 per 10 person-years, while other tumor reduction procedures were also observed including nephrectomy (0.14 per 10 person-years) and laser therapy to the retina (0.36 per 10 person-years), albeit less commonly.

**Table 2:** Treatment patterns for patients with VHL-CNS-Hb

CNS-Hb-related Treatments^1^	Unadjusted Incidence Rates for VHL-CNS-Hb Cohort	
	(*N* = 220)	
	Total Events	Incidence Rate (95% CI) Events per 10 Person-Years
Nephrectomy	9	0.14 (0.07, 0.26)
Renal ablation	5	0.08 (0.03, 0.17)
Radiotherapy	27	0.42 (0.28, 0.60)
Targeted therapies for RCC	150	2.34 (1.99, 2.74)
Axitinib	15	0.23 (0.14, 0.38)
Bevacizumab	47	0.73 (0.55, 0.97)
Cabozantinib	21	0.33 (0.21, 0.49)
Everolimus	10	0.16 (0.08, 0.28)
Lenvatinib	4	0.06 (0.02, 0.15)
Nivolumab	5	0.08 (0.03, 0.17)
Pazopanib	40	0.62 (0.45, 0.84)
Pembrolizumab	4	0.06 (0.02, 0.15)
Sunitinib	4	0.06 (0.02, 0.15)
Laser therapy to retina	23	0.36 (0.23, 0.53)
Surgical removal of cerebellar and spinal Hbs	33	0.52 (0.36, 0.71)
Surgical removal of adrenal pheochromocytomas	3	0.05 (0.01, 0.12)
Surgical removal of pancreatic neuroendocrine tumors	2	0.03 (0.01, 0.1)

**Abbreviations**: CI: confidence interval; CNS: central nervous system; GEE: generalized estimating equation; Hb: hemangioblastoma; RCC: renal cell carcinoma; VHL: von Hippel-Lindau

**Note:**

[1] Mean follow-up time was 34.9 months for patients with VHL-CNS-Hb, with a total of 640 person-years

### Pain management drug use

**Fig. 2:**
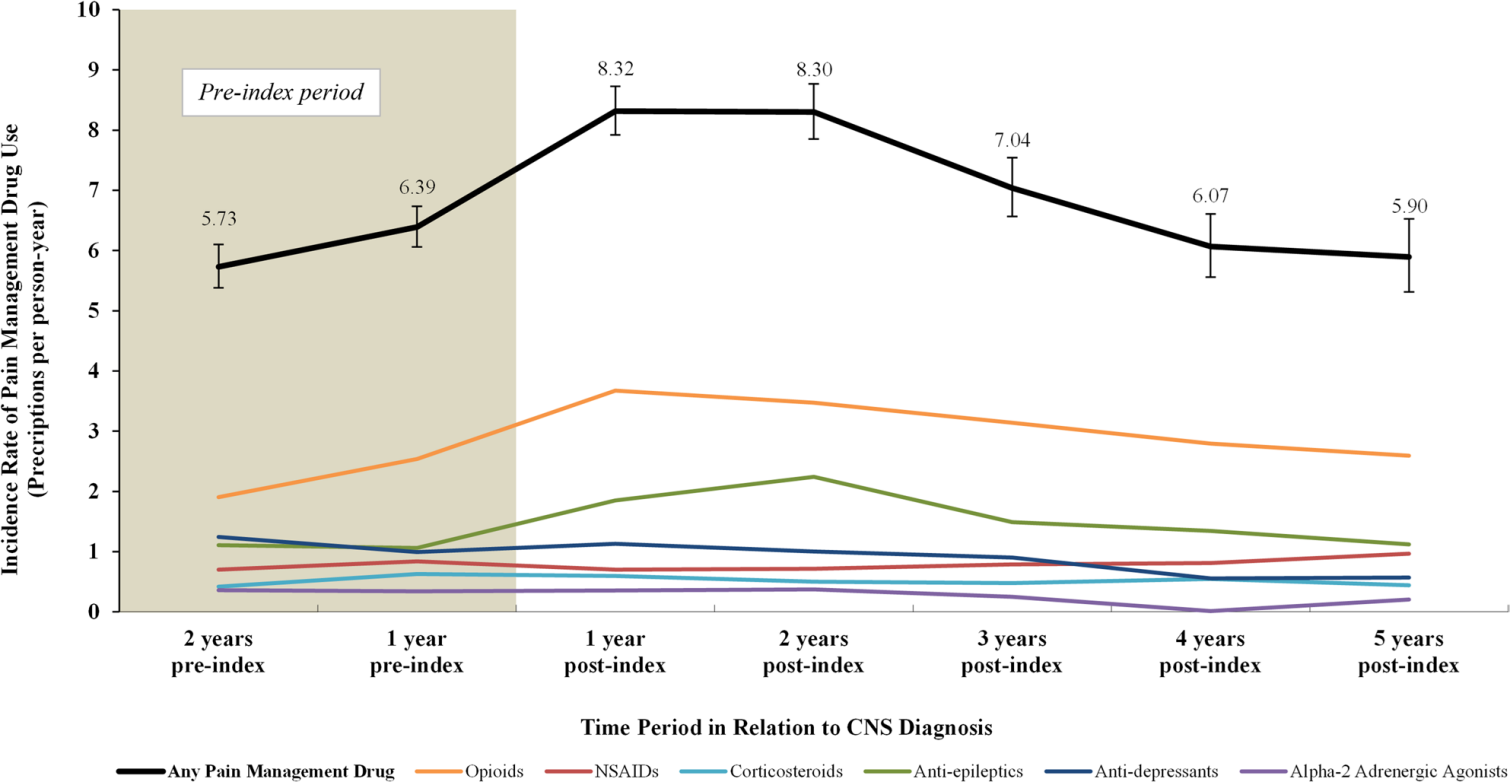
Pain management drug use over time among patients with VHL-CNS-Hb. **Abbreviations:** CNS: central nervous system; Hb: hemangioblastoma; NSAID: Non-steroidal anti-inflammatory drug; VHL: von Hippel-Lindau. **Notes:** [1] The index visit was considered within the “1 year post-index” time period. [2] Error bars indicate upper and lower bounds of the 95% confidence interval (Byar’s approximation)

During the assessment period from 2 years pre-index to 5 years post index, the unadjusted incidence rate of any pain management drug use was highest in the first two years post index (8.30–8.32 prescriptions per person-year) and decreased thereafter (Fig. 2). Among the pain management drugs of interest, opioids had the highest rates of use over the years, with a peak at 1 year post index of 3.67 prescription per person-year. Aside from anti-epileptics, which peaked at 2 years post index, use of other pain management drugs remained relatively stable over time.

During the study period, patients in the VHL-CNS-Hb cohort had higher rates of any pain management drug use compared to patients in the control cohort (adjusted IRR: 1.96 [95% CI: 1.45, 2.64]; *P* < 0.001; Table 3). More specifically, opioids (2.35 [1.54, 3.58]; *P* < 0.001), corticosteroids (1.55 [1.15, 2.09]; *P* = 0.004), and anti-epileptics (3.40 [1.99, 5.82]; *P* < 0.001) were more commonly used in the VHL-CNS-Hb than control cohort after adjusting for baseline covariates. Given the small sample sizes, models for cannabinoids and botulinum toxin were not fit.

**Table 3:** Comparison of pain management drug use, monitoring procedures, and medical specialist visits

	Incidence Rates [Per-Person Year]				IRR [VHL-CNS-Hb vs. Control]					
	Unadjusted		Adjusted		Unadjusted			Adjusted		
	VHL-CNS-Hb (*N* = 220)	Controls (*N* = 1,100)	VHL-CNS-Hb (*N* = 220)	Controls (*N* = 1,100)	IRR (95% CI)	*P*-value		IRR (95% CI)	*P*-value	
***Any Pain Management Drug***	***8.15***	***3.29***	***5.28***	***2.70***	***2.48 (1.93***,*** 3.19)***	***< 0.001***	*	***1.96 (1.45***,*** 2.64)***	***< 0.001***	*
Opioids	3.52	1.28	2.35	1.00	2.75 (1.93, 3.91)	< 0.001	*	2.35 (1.54, 3.58)	< 0.001	*
NSAIDs	0.72	0.63	0.57	0.61	1.15 (0.79, 1.66)	0.468		0.94 (0.64, 1.38)	0.748	
Corticosteroids	0.63	0.30	0.45	0.29	2.08 (1.55, 2.79)	< 0.001	*	1.55 (1.15, 2.09)	0.004	*
Anti-epileptics	1.85	0.46	0.95	0.28	3.99 (2.81, 5.68)	< 0.001	*	3.40 (1.99, 5.82)	< 0.001	*
Anti-depressants	1.00	0.44	0.46	0.26	2.26 (1.35, 3.76)	0.002	*	1.77 (0.88, 3.58)	0.112	
Alpha-2 adrenergic agonists	0.34	0.13	0.18	0.12	2.54 (0.98, 6.58)	0.055		1.47 (0.59, 3.66)	0.413	
***Any Monitoring Procedure***	***9.02***	***2.09***	***7.80***	***1.99***	***4.32 (3.54***,*** 5.28)***	***< 0.001***	*	***3.93 (3.15***,*** 4.90)***	***< 0.001***	*
Ultrasound	0.45	0.18	0.41	0.18	2.48 (1.94, 3.15)	< 0.001	*	2.32 (1.80, 2.98)	< 0.001	*
MRI	0.98	0.11	0.85	0.11	9.28 (7.35, 11.72)	< 0.001	*	8.07 (6.30, 10.34)	< 0.001	*
CT Scans	2.22	0.25	1.81	0.23	8.82 (6.10, 12.77)	< 0.001	*	7.81 (5.41, 11.28)	< 0.001	*
Chest X-ray	1.79	0.38	1.52	0.32	4.77 (3.12, 7.29)	< 0.001	*	4.81 (2.96, 7.83)	< 0.001	*
Retinal Exams	0.34	0.09	0.33	0.09	3.58 (2.22, 5.75)	< 0.001	*	3.73 (2.17, 6.43)	< 0.001	*
Metanephrines^1^	0.12	0.00	0.08	0.00	249.69 (34.08, 1,829.19)	< 0.001	*	230.08 (30.40, 1,741.62)	< 0.001	*
Renal Function Tests	2.79	1.03	2.38	1.00	2.72 (2.27, 3.25)	< 0.001	*	2.39 (1.98, 2.88)	< 0.001	*
***Medical Specialist Visits***										
Oncology	1.72	0.22	1.03	0.14	7.92 (4.51, 13.91)	< 0.001	*	7.47 (4.19, 13.29)	< 0.001	*
Urology	0.53	0.14	0.38	0.12	3.74 (2.41, 5.83)	< 0.001	*	3.27 (1.96, 5.46)	< 0.001	*
Nephrology	0.70	0.06	0.72	0.04	12.66 (6.26, 25.61)	< 0.001	*	20.30 (6.37, 64.68)	< 0.001	*
Genetics	0.03	0.00	0.02	0.00	16.53 (4.23, 64.54)	< 0.001	*	12.01 (3.29, 43.84)	< 0.001	*
Neurosurgery	0.70	0.03	0.66	0.03	25.46 (15.02, 43.16)	< 0.001	*	25.44 (14.34, 45.14)	< 0.001	*
Ophthalmology	0.64	0.22	0.54	0.21	2.93 (1.99, 4.34)	< 0.001	*	2.59 (1.68, 3.99)	< 0.001	*
Endocrinology	0.24	0.09	0.20	0.08	2.60 (1.59, 4.24)	< 0.001	*	2.49 (1.43, 4.31)	0.001	*

**Abbreviations**: CI: confidence interval; CNS: central nervous system; CT: computed tomography; GEE: generalized estimating equation; Hb: hemangioblastoma; IRR: incidence rate ratio; MRI: magnetic resonance imaging; NSAID: non-steroidal anti-inflammatory drug; VHL: von Hippel-Lindau

**Notes**:

* denotes P-value < 0.05

[1] Given the low incidence rates of metanephrine use in both cohorts, this GEE model was adjusted for age at index and sex only

### Disease monitoring procedures

**Fig. 3:**
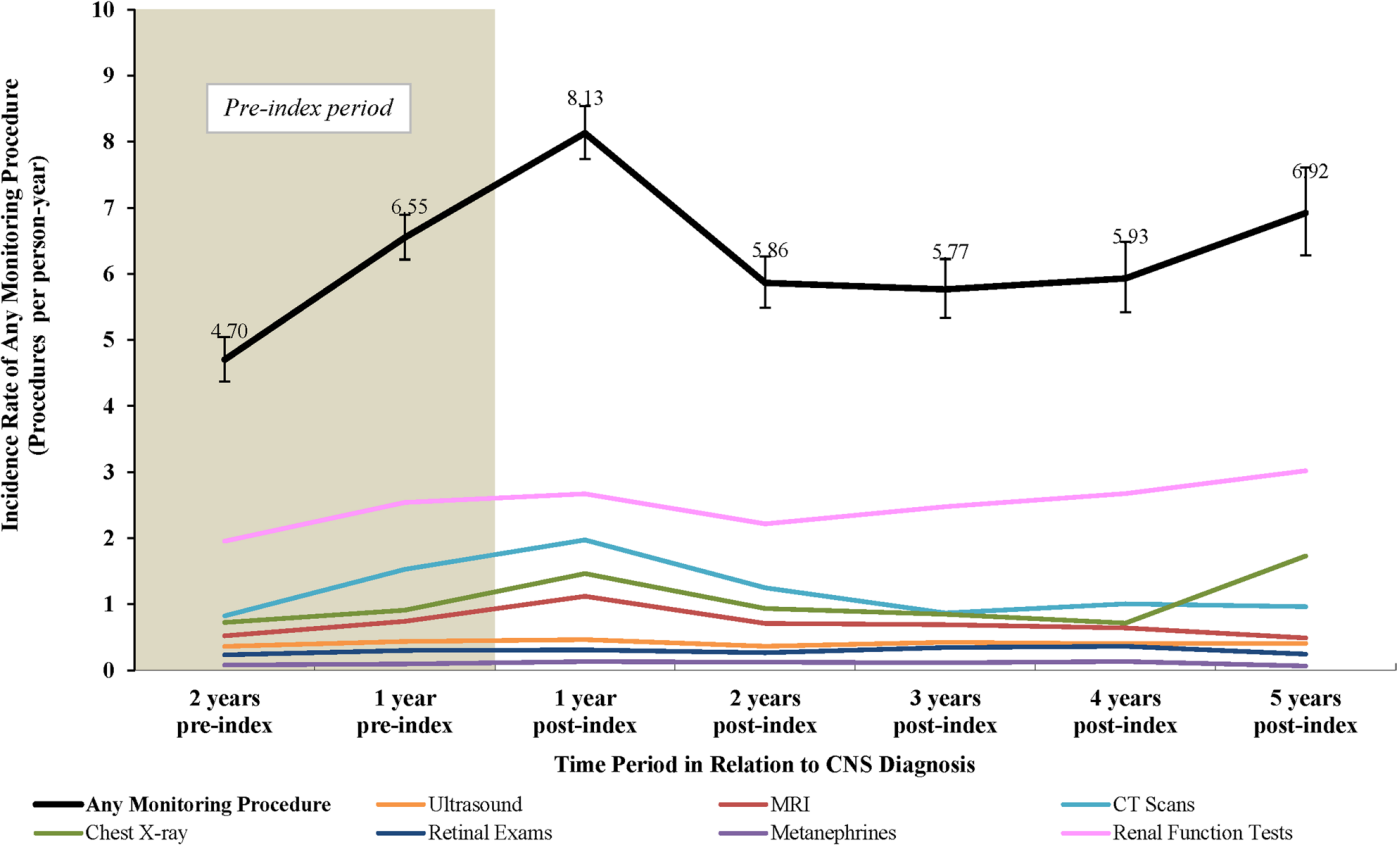
VHL and CNS monitoring procedures over time among patients with VHL-CNS-Hb. **Abbreviations:** CNS: central nervous system; CT: computed tomography; Hb: hemangioblastoma; MRI: magnetic resonance imaging; VHL: von Hippel-Lindau. **Note**: [1] The index visit was considered within the “1 year post-index” time period.

During the assessment period from 2 years pre-index to 5 years post index, the unadjusted incidence rate of any disease monitoring procedure was highest in the first year post index (8.13 procedures per person-year; Fig. 3). Among the monitoring procedures of interest, renal function tests were the most frequently observed over the years, with an incidence rate at 1 year post index of 2.67 procedures per person-year and a peak at 5 years post index of 3.02 procedures per person-year.

During the study period, patients in the VHL-CNS-Hb cohort had higher rates of any disease monitoring procedure compared with patients in the control cohort (adjusted IRR: 3.93 [95% CI: 3.15, 4.90]; *P* < 0.001; Table 3). Incidence rates for individual monitoring procedures were also significantly higher in the VHL-CNS-Hb cohort compared with the control cohort after adjusting for baseline covariates (all *P* < 0.001).

### Medical specialist visits

**Fig. 4:**
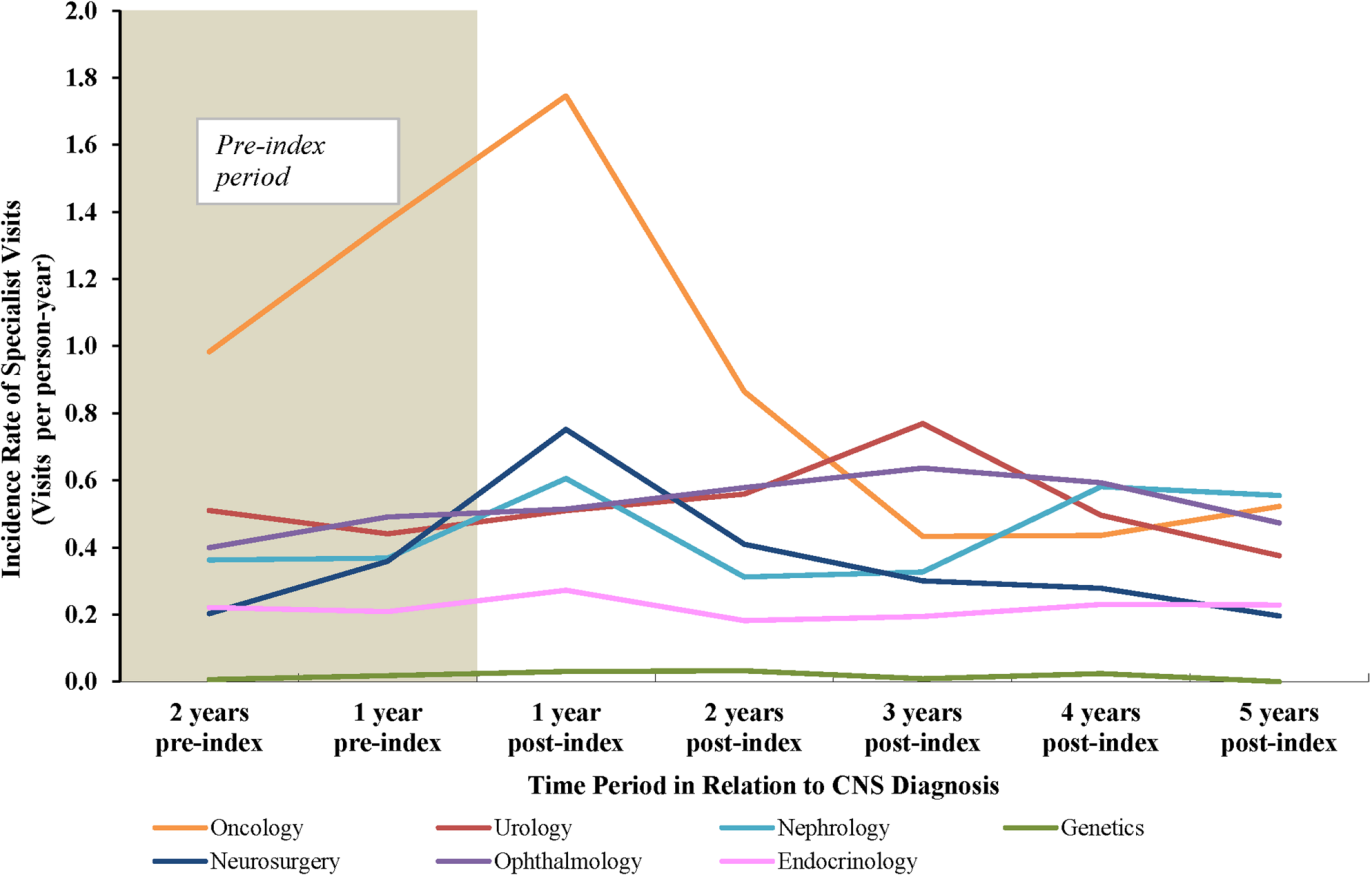
Medical specialist visits over time among patients with VHL-CNS-Hb. **Abbreviations**: CNS: central nervous system; Hb: hemangioblastoma; VHL: von Hippel-Lindau. **Notes:** [1] Visits with specialists were identified from claims associated with relevant Healthcare Provider Taxonomy codes. Claims that occurred in direct succession (i.e., back-to-back days) with the same specialist were considered as one visit. [2] The index visit was considered within the “1 year post-index” time period

In the first year post index, medical specialist visits were most commonly for oncology (1.75 per person-year) visits (Fig. 4). Unadjusted incidence rates were also elevated for neurosurgery (0.75 per person-year) and nephrology (0.61 per person-year) visits at this time. During the assessment period from 2 years pre-index to 5 years post index, the rates of these three specialist visits peaked in the first-year post index and then generally declined over time. The rates of ophthalmology and urology visits tended to increase slightly during the post-index period, while the rate of endocrinology and genetics visits remained relatively stable over time.

During the study period, patients in the VHL-CNS-Hb cohort had higher rates of all specialist visits compared with the control cohort after adjusting for baseline covariates (all *P* ≤ 0.001; Table 3).

## Discussion

This real-world study leveraged the use of a recently developed and validated claims-based algorithm to identify patients with VHL-CNS-Hb and compare their clinical burden relative to those without VHL disease or CNS-Hb in the US. Of note, targeted therapy for RCC was commonly observed among this cohort of patients with VHL-CNS-Hb, along with nephrectomy and laser therapy to the retina, suggesting that many patients with VHL-CNS-Hb had concurrent VHL-associated tumors elsewhere in the body. Indeed, 40.0% of patients receiving radiotherapy during the study period had diagnosis codes indicating RCC at some point in their medical claims history. Pain management drug use was more common among patients with VHL-CNS-Hb than matched controls, especially opioids and anti-epileptics. Similarly, patients with VHL-CNS-Hb had more frequent disease monitoring than those without VHL or CNS-Hb, with renal function tests being particularly common. Lastly, patients with VHL-CNS-Hb had a multitude of medical visits with different specialists during the years before and after CNS-Hb diagnosis, highlighting the multifaceted and complex nature of VHL disease, requiring regular care from a multidisciplinary team.

To our knowledge, this is the first study to assess the treatment and disease monitoring patterns and specialist visits of patients with VHL-CNS-Hb in real-world clinical practice. One prior analysis of the US National Cancer Database by Huang et al. described the treatment patterns of patients with CNS-Hb in general and found that surgery (mainly gross total resection) was the most common treatment option, but other outcomes like disease monitoring and specialist visits were not evaluated [10]. As such, the current study builds upon this prior analysis and represents an important first account of the substantial clinical burden associated with VHL-CNS-Hb, thus contributing important insight to the limited existing literature on VHL disease.

The findings of this study complement a prior analysis by Jonasch et al. describing the treatment patterns of patients with VHL-RCC, which demonstrated a similarly large management and disease monitoring burden associated with VHL manifestations [12]. Notably, patients with VHL-RCC had non-negligible rates of tumor reduction procedures targeting non-RCC neoplasms (e.g., retinal and CNS-Hb) in the prior study [12], consistent with our finding of high rates of RCC targeted therapy use among patients with VHL-CNS-Hb. This observation suggests that patients with VHL disease may have multiple VHL manifestations throughout life, with a particularly large overlap between CNS-Hb and RCC. Indeed, one way to diagnose VHL disease without a family history is by the presence of one CNS-Hb plus a visceral (e.g., RCC) or endolymphatic sac tumor [8]. Furthermore, in the pivotal clinical trial of belzutifan for the treatment of VHL-RCC, 82% of patients with VHL-RCC also had VHL-CNS-Hb during the baseline period [13]. Therefore, the need for multiple tumor reduction procedures targeting different VHL manifestations demonstrated in this and the prior study by Jonasch et al. further highlights the long-term morbidity and multifaceted treatment burden of patients with VHL disease.

In addition to the clinical burden, management of VHL-CNS-Hb has previously been shown to be associated with high costs as well [9]. In a separate study by Jonasch et al., patients with VHL-CNS-Hb had significantly more inpatient, outpatient, and emergency department visits compared with controls without VHL disease or CNS-Hb, resulting in $49,645 higher annual healthcare costs [9]. Notably, among patients who received surgery, average hospitalization costs were highest for those with surgical removal of CNS-Hb [9]. While the current study did not evaluate healthcare costs, it provides important clinical context regarding the tumor reduction procedures, frequent disease monitoring, and multitude of medical specialist visits required over the course of the patient journey, which likely contribute to the substantial economic burden observed in the prior analysis.

Given the need for regular invasive surgeries and healthcare services throughout a patient’s lifetime, the use of effective systemic therapy for VHL disease may help to reduce this clinical burden [7]. The HIF-2α inhibitor belzutifan is the first systemic therapy approved by the Food and Drug Administration (FDA) for the treatment of patients with VHL disease who require therapy for RCC, CNS-Hb, or pancreatic neuroendocrine tumors, not requiring immediate surgery [14]. In the subgroup analysis of the pivotal LITESPARK-004 clinical trial, consistent and durable antitumor activity was observed with belzutifan treatment among patients with VHL-CNS-Hb [15]. Similarly, belzutifan was associated with strong disease responses and was well-tolerated in two small real-world studies of patients with CNS-Hb [16, 17]. However, since the approval of belzutifan occurred after the data cut of the current study, further research is warranted to determine if this clinical effectiveness translates to reduced surgeries and healthcare service use in real-world clinical practice.

### Limitations

The study findings should be interpreted within the context of some limitations. Patients with VHL disease were identified based on a claims-based algorithm [2, 9], which relied on the accuracy and completeness of available physician notes and claims data. Additionally, VHL disease was defined based on diagnoses of manifestations that may also occur sporadically, without being associated with VHL disease; as such, some patients may have been misclassified by the algorithm.

The index date was defined as the first observed CNS-Hb diagnosis for patients in the VHL-CNS-Hb cohort. Diagnosis-driven index dates may coincide with periods of increased healthcare utilization related to diagnostic evaluation and/or treatment initiation before and after index. Because control patients had neither VHL nor CNS-Hb, there was no clinically meaningful diagnosis date on which to anchor an index date; therefore, a medical claim date was randomly selected as the index date for control patients, a common practice in claims-based analyses [18, 19]. Although continuous enrollment and follow-up windows were defined identically across cohorts, healthcare utilization patterns around randomly selected dates may differ systematically from patterns around diagnosis-driven index dates. As a result, short-term differences in pain management drug use, disease monitoring procedures, and medical specialist visits between the cohorts may partially reflect heightened healthcare utilization around the VHL-CNS-Hb diagnosis date. However, given the extended follow-up duration in both cohorts (34.9 months for patients with VHL-CNS-Hb, 24.2 months for the control cohort), any such short-term differences are likely to be attenuated.

Despite the use of propensity score matching to balance the two study cohorts, confounding may have occurred due to unmeasured or unavailable covariates. Lastly, as the study was conducted among commercially insured/Medicare Advantage patients with VHL disease, the results may not be generalizable to those with other or no insurance coverage.

## Conclusions

In this real-world study, patients with VHL-CNS-Hb harbored a substantial, long-term clinical burden that included treatment for multiple VHL manifestations, frequent pain management drug use, and high levels of healthcare use for disease monitoring and specialist visits. These findings highlight the multifaceted and complex nature of VHL disease management and the opportunity for effective tumor control to potentially reduce this burden and patient morbidity.

## Data Availability

The data that support the findings of this study are available from Optum, but restrictions apply to the availability of these data, which were used under license for the current study, and so are not publicly available. Any researchers interested in obtaining the data used in this study can access the database through Optum, under a license agreement, including the payment of appropriate license fee.

## Declarations

### Previous presentations

Part of the material in this manuscript was presented at the American Society of Clinical Oncology (ASCO) 2024 Annual Meeting held May 31 – June 4, 2024, in Chicago, Illinois, United States, and online as a poster presentation.

## Abbreviations

CCI: Charlson Comorbidity Index
CDM: Clinformatics^®^ Data Mart Database
CI: Confidence interval
CNS: Central nervous system
CT: Computerized tomography
EPO: Exclusive provider organization
FDA: Food and Drug Administration
GEE: Generalized estimating equation
Hb: Hemangioblastoma
HMO: Health maintenance organization
ICD: International Classification of Diseases
IRR: Incidence rate ratio
MRI: Magnetic resonance imaging
NSAID: Non-steroidal anti-inflammatory drug
POS: Point of service
PPO: Preferred provider organization
RCC: Renal cell carcinoma
SD: Standard deviation
US: United States
USD: United States dollar
VHL: Von Hippel-Lindau
